# Orchid Flora of the Guelma Region (North-Eastern Algeria), a Little-Known Group for Algerian Flora

**DOI:** 10.3390/plants14243833

**Published:** 2025-12-16

**Authors:** Kenza Tebani, Ángel Enrique Salvo-Tierra, Jaime F. Pereña-Ortiz, Lamia Boutabia, Tarek Hamel, Gérard de Belair, Amel Meddad-Hamza, Salah Telailia

**Affiliations:** 1 Agriculture and Ecosystem Functioning Laboratory, Faculty of Natural and Life Sciences, Chadli Bendjedid University, El Tarf 36000, Algeria; kenzagro24@gmail.com (K.T.); boutabia-lamia@univ-eltarf.dz (L.B.); tarek_hamel@yahoo.fr (T.H.); telailia-salah@univ-eltarf.dz (S.T.); 2 Department of Botany and Plant Physiology, Faculty of Science, University of Málaga, Bulevard Louis Pasteur, 29010 Málaga, Spain; salvo@uma.es; 3 Department of Biology, Faculty of Sciences, Badji Mokhtar University, Annaba 23000, Algeria; gerarddebelair@gmail.com (G.d.B.); amel_meddad@yahoo.fr (A.M.-H.)

**Keywords:** orchid flora, Guelma province, northeastern Algeria, *Ophrys*, endemism, OBUs, conservation

## Abstract

Knowledge of Algeria’s orchid flora has increased considerably over the past two decades; however, certain regions, such as Guelma Province in northeastern Algeria, remain poorly studied. Between 2013 and 2024, survey work was conducted in this region using a subjective sampling approach. A total of 40 stations were inventoried, and ecological variables such as altitude, exposure, and vegetation cover were recorded to interpret the distribution patterns of orchid taxa. In total, 37 taxa including 16 species, 19 subspecies and 2 hybrids were identified, with a predominance of the genus *Ophrys* (19 taxa). Among these, ten taxa exhibit a close endemic relationship with neighboring North African territories, and 22 are classified as rare in Algeria. Several taxa also appear as widespread and abundant, enriching the known orchid flora of the study area. Multivariate analyses revealed site typologies and environmental variables influencing the distribution of the recorded species. Cluster analysis identified five distinct Operational Biogeographical Units (OBUs), corresponding to specific environmental characteristics and orchid physiognomies. Furthermore, correlations between the studied taxa and environmental factors suggest that their occurrence is strongly influenced by these variables. Given the high vulnerability of the surveyed sites and the increasing anthropogenic pressures they face, the implementation of urgent conservation measures to protect these habitats and their components is strongly recommended.

## 1. Introduction

The knowledge, characterization, classification, and conservation of taxa constitute a global scientific priority for the assessment and management of biodiversity [1]. Efforts to study the flora are essential for understanding the fundamental biological traits of plants [2]. Comprehensive information on the biology and ecology of species is critical for effective biodiversity conservation and management [3]. It is well established that the spatial distribution of abiotic and biotic factors plays a key role in shaping the distribution, abundance, and composition of plant species [4].

The Mediterranean region is widely recognized as one of the world’s principal hotspots of plant biodiversity [5], ranking as the third richest global hotspot in terms of plant diversity [6]. This region represents a major biogeographical crossroads, encompassing Euro-Siberian, Mediterranean, and subtropical floristic elements [7,8].

In North Africa and Europe, orchids occur across a wide range of ecosystems, including forests, scrublands, meadows, moorlands, peat bogs, and marshes [9,10,11,12,13]. Numerous studies have demonstrated that orchid species richness, distribution, abundance, growth, and reproduction are influenced by a variety of ecological factors, including vegetation type, geological substrate, soil properties, climate, latitude, exposure, and temperature [14,15,16,17].

Despite extensive research on European orchids [18,19,20,21,22,23], comparatively little is known about the orchid flora of the southern Mediterranean coastline. In particular, recent studies on Algerian orchids remain limited. Dobignard and Chatelain (2010) [24] reported a total of 75 orchid taxa for Algeria. Additional contributions include studies by de Bélair (2000) [25], de Bélair et al. (2005) [26], Hamel and Meddad-Hamza (2016) [27], Hamel et al. (2017) [28], Boukehili et al. (2018) [29], and Boutabia et al. (2019) [30], which primarily focus on the Numidia region. The foundational study by de Bélair et al. (2005) [26] on the orchids of Numidia—including the northern part of Guelma Province—and its later extension by Hamel et al. (2018) [31] for the Djebel Taya area (southwest of Guelma), did not cover the entire province, particularly its southern and eastern sectors.

Moreover, existing bibliographic records provide neither quantitative estimates of orchid populations nor detailed information on the precise locations and ecological characteristics of each taxon. The orchids of the mountainous region of Guelma are currently subject to considerable anthropogenic pressures and are exposed to climatic constraints associated with their position at the southern limit of their distribution range, adjacent to semi-arid conditions—factors that considerably increase their vulnerability. As a result, these habitats face the risk of disappearing before they can be fully documented and understood.

Given these knowledge gaps regarding orchid diversity in the region, this study seeks to address the following questions: (1) What orchid taxa are present in Guelma Province? (2) In which habitats do these taxa occur? (3) What are the current population sizes of the recorded taxa? (4) Which ecological factors influence their distribution?

## 2. Materials and Methods

### 2.1. Study Area

**Figure 1:**
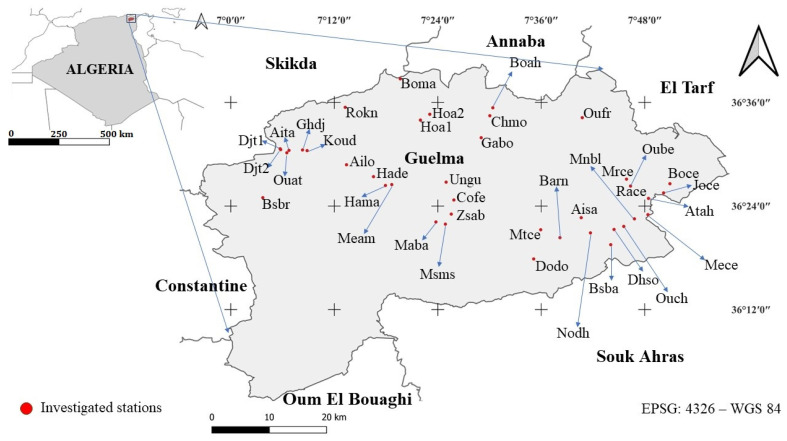
Geographical location of the Guelma province and studied stations. Map represented in coordinate reference system: EPSG 4326-WGS 84.

Located in northeastern Algeria and covering a total area of 3686.84 km^2^, the Guelma province is bordered to the south by Oum El Bouaghi, to the north by Annaba, to the east by El Tarf and Souk Ahras, and to the west by Skikda and Constantine [32] (Figure 1). Climatically, the region lies between the sub-humid zone of coastal Numidia (Skikda–Annaba–El Kala) and the semi-arid zone extending through Constantine, Oum El Bouaghi, and Tébessa [33]. Geologically, it is characterized by travertine formations rich in aragonite, calcite, and siliceous jasper, overlying Pliocene clay substrates [34]. This region lies in a transitional zone between the Numidian subsector (K_3_) and the Tell Constantinois (C_1_), according to the biogeographical subdivision proposed by Quézel and Santa (1962; 1963) [35]. is recognized as one of the most important plant areas in Algeria [36,37,38], along with the Great and Small Kabylia sectors (K_1_ and K_2_). These areas exhibit the highest biodiversity index on a national scale [7].

The main natural plant formations in the province are holm oak forests, with a predominance of cork oak (*Quercus suber* L.) and zeen oak (*Quercus canariensis* Willd.), accompanied by scrubland dominated by wild olive (*Olea europaea* L.) and mastic (*Pistacia lentiscus* L.) [39].

A total of 40 stations were surveyed in the Guelma province (Table S1), mainly in cemeteries and transitional zones. These included natural grassland formations, field margins, pond edges, roadside verges, and other natural landscape features of ecological interest.

### 2.2. Floristic Study

Due to the lack of information on the distribution of orchids in the Guelma province, a subjective sampling method was adopted, focused directly on known orchid locations [40], particularly cemeteries, as previously applied by de Bélair et al. (2005) [26] in Numidia and by Nagy et al. (2025) [41] in Europe. Field surveys were conducted regularly in both space and time throughout the flowering period (September–June) during the years 2013–2024.

At each surveyed site, the presence of taxa was recorded, and geographical coordinates, altitude, exposure, slope, and substrate characteristics [42], as well as grazing pressure, were noted. Additional ecological parameters were measured, including orchidological surface area and coverage indices for woody and herbaceous taxa (Table S2).

Taxa were identified using the floras of Maire (1960) [43], Quézel & Santa (1962) [35], Flora Vascular de Andalucía Oriental [44], and more recent works by Martin et al. (2015, 2020) [11,45]. Nomenclature was standardized according to the synonymy index of Dobignard & Chatelain (2010) [24] and its updated online version. Chorological data were established based on these same references.

The chorological types of the recorded taxa followed the classifications of Pignatti (1982) [46], Blanca et al. (2009) [44], and Jeanmonod & Gamisans (2013) [47]. Information regarding endemic taxa was sourced from Dobignard & Chatelain (2010) [24].

Taxa of heritage value as all endemic or sub-endemic taxa were defined, as well as any taxa classified in the reference flora of Quézel & Santa (1962) [35] or in previous studies on Algerian orchids [11,26,29] as fairly rare, rare, or very rare, along with the authors’ own field observations. Vulnerability criteria were also considered at a global scale, as defined by the International Union for Conservation of Nature [48] and the IUCN Red List at global and/or Mediterranean levels [49]. The classification categories applied followed the IUCN Red List version 3.1 criteria [49].

The resulting Red List allowed the identification of taxa at highest risk of extinction and the definition of conservation priorities for plant biodiversity protection. Protection status at the national level was assessed based on the official list of non-cultivated plant taxa protected in Algeria under Decree No. 03-12/12-28 [50].

The field data collected were compiled into a Basic Data Matrix (BDM) and validated by cross-checking results using the Menhinick index and Fisher’s logarithmic series model [34]. The Menhinick index compares floristic diversity between localities by considering the relationship between taxa richness and sample size. It is often combined with other biodiversity indices to achieve a more complete understanding of community composition and structure. Fisher’s logarithmic series model [51] was applied to describe the relationship between the number of taxa and the number of individuals per taxa mathematically.

### 2.3. Numerical Analysis of Floristic Data

The Outlying Mean Index (OMI) analysis [52] was employed to assess how orchid composition (expressed on a four-point ordinal scale) is influenced by environmental variables. Two datasets were used: one containing species occurrence data and the other comprising the explanatory environmental variables.

The first output of the OMI analysis quantifies the marginality of each taxon’s habitat distribution, defined as the distance between the mean habitat conditions used by a taxon and the mean habitat conditions across the entire study area. The second output provides a measure of niche breadth (taxon tolerance), representing the range of environmental conditions occupied by each taxon along the sampled environmental gradients.

In this framework, high tolerance values indicate generalist taxa, occurring across habitats characterized by broad environmental variation, whereas low tolerance values identify specialist taxa, restricted to a narrow range of habitat conditions. The statistical significance of each taxon’s marginality was assessed using a random permutation test.

The OMI analysis also provides a residual tolerance value, which reflects the extent to which the selected environmental variables explain each taxon’s ecological niche.

All analyses were conducted in the R programming environment (version 3.0.1) [53], using the ade4 package [54] for multivariate analysis.

To further examine the relationships among taxa, their territorial affinities, and the ecological variables, a clustering analysis was performed on the localities (referred to as Operational Geographical Units, OGUs) based on similarities in floristic composition. This approach enabled the delineation of Operational Biogeographical Units (OBUs) derived from the clustering results. Ward’s method was selected because it is considered one of the most statistically robust hierarchical clustering techniques. All clustering procedures and subsequent analyses were conducted using PAST v.4.17c software [55].

A Principal Component Analysis (PCA) was also conducted to visualize and interpret the relationships between taxa and the environmental gradients. A network plot linking principal components (PCs) and taxa was generated using the Bipartite Linear Algorithm, enabling the identification of those taxa exerting the strongest influence on variance in PC1, PC2, and PC3.

Finally, a conditioning weight index (Conditional Value -CV-) was proposed to evaluate the relative importance of the highest-ranked taxa, as defined in Equation (1). This index is based on the weight of each taxon in the PCA relative to the percentage of variance (%var) explained by the corresponding principal component.

CVtaxa n = Σ [(PC1*%vartaxa n) + (PC2*%vartaxa n) + (PC3*%vartaxa n)](1)

In a second step, eco-chorotypes were defined based on the similarity of orchid-floristic distributions and the nine environmental variables considered. Ward’s method was applied to calculate the mean representation of each chorotype within the different Operational Biogeographical Units (OBUs) and, conversely, the mean representation of each OBU within the defined chorotypes.

Concurrently, a set of indicator taxa was identified through correlation analyses (Pearson’s linear r) between taxa and ecological variables, as well as between OBUs and ecological variables.

Finally, a double-entry matrix of chorotypes and OBUs was constructed, allowing the calculation of correlations between these two grouping types and the identification of the most distinctive chorotypes characterizing each OBU.

## 3. Results

### 3.1. Orchid Diversity

Thirty-seven orchid taxa, including 16 species, 19 subspecies and 2 hybrids were recorded in Guelma Province (Appendix A), representing eleven genera. The genus *Ophrys* was the most diverse, with nineteen taxa.

Several *Ophrys* taxa were present at all study sites, including *Ophrys bombyliflora*, *O. lutea* subsp. *lutea*, *O. speculum* subsp. *speculum*, and *Serapias parviflora*. Some taxa showed notably high abundance, such as *Orchis italica* and *Ophrys lutea* subsp. *lutea*, each with about 200 individuals. In contrast, several species were rare within the study area, including *Cephalanthera longifolia* (1 individual), *Neotinea maculata* and *Spiranthes spiralis* (5 individuals each), *Limodorum abortivum* (7 individuals), *Neotinea lactea* (9 individuals), *Serapias lingua* subsp. *lingua* (25 individuals), and both *S. lingua* subsp. *stenopetala* and *S. lingua* subsp. *tunetana* (10 individuals each) (Table S3).

**Figure 2:**
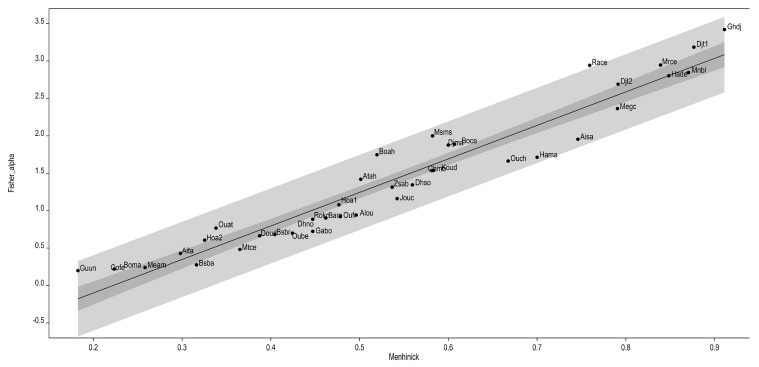
Validation of sampling by estimating the diversity correspondence between the Menhinick index (Axis X) and Fisher’s log series model (Axis Y). The lighter band corresponds to the distribution range limits, and the darker band to the 95% confidence limits. The order generated responds to the increase in diversity, which is very high in Ghdj, Djt1, Mrce, Mnbl and Hadc.

The logarithmic-series model provided a good fit to the data, reflecting assemblages characterized by a few abundant species and many rare ones, consistent with the observed patterns. This model is particularly suitable when one or more environmental factors exert a strong influence on species composition. As illustrated in Figure 2, the samples collected at each site were sufficiently representative of the orchid communities in the study area.

Regarding the habitats hosting these taxa, low scrubland dominated by wild olive (*Olea europaea*) and mastic trees (*Pistacia lentiscus*), as well as wetlands, showed the highest orchid diversity, with approximately eight different taxa each. Medium scrubland exhibited the lowest diversity, supporting only one to three taxa.

The highest orchid abundance was recorded in riparian areas, covering more than 1000 m^2^ with the presence of several taxa, followed by low scrub cemeteries, with over 700 m^2^ of orchid cover. Medium and tall scrublands showed intermediate values, with approximately 300 m^2^ and 500 m^2^, respectively. Cork oak (*Quercus suber*) woodland presented the lowest abundance, with an orchid cover area of less than 100 m^2^.

### 3.2. Chorological Diversity and Heritage Value

According to available bibliographic information, the recorded taxa belong to three chorological groups (Table S4):

**Mediterranean orchid group**: This group dominates, comprising 26 taxa (70.27% of the recorded flora), including 25 Mediterranean (*sensu stricto*) taxa and one Atlantic–Mediterranean taxa.

**Northern orchid group**: Represented by a single European taxon, *Anacamptis fragrans*.

**Endemic orchid group**: Consisting of 10 taxa (27.02% of the studied orchid flora). This group includes six taxa endemic to Algeria and Tunisia, one taxon endemic to Algeria (*Ophrys fusca* subsp. *maghrebiaca)*, one Betic–Maghrebian endemic (*Ophrys atlantica*), one endemic to Algeria, Tunisia, and Italy (*Orchis patens*), and one restricted to Algeria, Tunisia, and Morocco (*Ophrys numida*).

The flora studied included 22 rare taxa, six of them classified as nationally protected (Table S3). This high number is attributed to habitat diversity, particularly in wild olive–mastic (*Olea europaea–Pistacia lentiscus*) scrublands, which host many rare and/or endemic taxa such as *Neotinea maculata* and *Ophrys marmorata* subsp. *marmorata*. Some are both endemic and rare, for example, *Orchis laeta*, *Dactylorhiza elata*, and *Serapias lingua* subsp. *stenopetala*.

### 3.3. Relationship Between Orchid Flora and Environmental Variables

**Figure 3:**
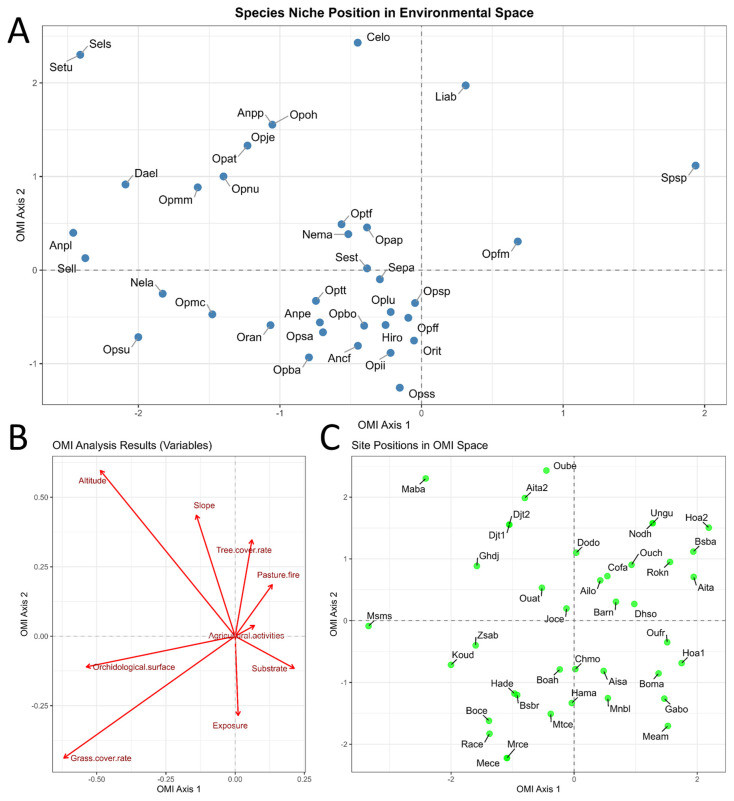
(**A**) Weighted positions of the species along the first two axes of the OMI analysis. (**B**) Canonical weights of the environmental variables. (**C**) Distribution of stations along the first two axes of the OMI analysis. The nomenclature of the points corresponds to the taxa in A and to the sites in C. The meaning can be consulted in the Supplementary Material.

The Outlying Mean Index (OMI) analysis examined the relationships among the 37 recorded orchid taxa (Figure 3A), the nine environmental variables (Figure 3B), and the 40 sampling stations (Figure 3C). The plane defined by the first two axes accounted for a total inertia of 61.22%. The results were statistically significant (*p* < 0.05), indicating that both station characteristics and environmental variables significantly influenced the heterogeneity in orchid abundance across the study area.

In the F1 plane (48.33% of the variance), the grouping of stations revealed a clear zonation of the orchid flora driven by key environmental factors. Based on the ecological affinities of the species, the first axis primarily reflects soil moisture. On the negative side, hygrophilous orchids such as *Dactylorhiza elata*, *Serapias lingua* subsp. *lingua*, *Orchis laeta*, *S. lingua* subsp. *stenopetala*, and *S. lingua* subsp. *tunetana* were positioned. In contrast, taxa associated with dry and degraded environments (*Spiranthes spiralis* and *Ophrys fusca* subsp. *maghrebiaca*) appeared on the positive side, corresponding to areas subject to intense pastoral activity.

Axis 2, which explained 12.89% of the total variance, distinguished high-altitude forest habitats from low-altitude preforest environments, suggesting an ecological gradient linked to vegetation closure. Stations such as Merdes Cemetery, El Megfel Cemetery, Medjez Amar, Galaat Bousbaa, and Menzel Bougataya Laïd were located at lower elevations (242–502 m) within semi-open preforest formations dominated by low and medium *Olea–Pistacia* scrublands. These conditions favored species such as *Ophrys tenthredinifera* subsp. *tenthredinifera*, *Ophrys scolopax* subsp. *scolopax*, and *Orchis italica*.

In contrast, high-altitude stations, including Ain Taya, Ouled Bechih, and Djebel Taya 1 and 2 (819–1043 m), corresponded to more closed forest formations, particularly cork oak (*Quercus suber*) forests and tall scrublands with *Crataegus azarolus* L. The characteristic orchids of these habitats (*Cephalanthera longifolia* and *Limodorum abortivum*) were typically associated with shaded, forested environments at higher elevations.

### 3.4. Definition of Operational Biogeographical Units (OBUs)

**Figure 4:**
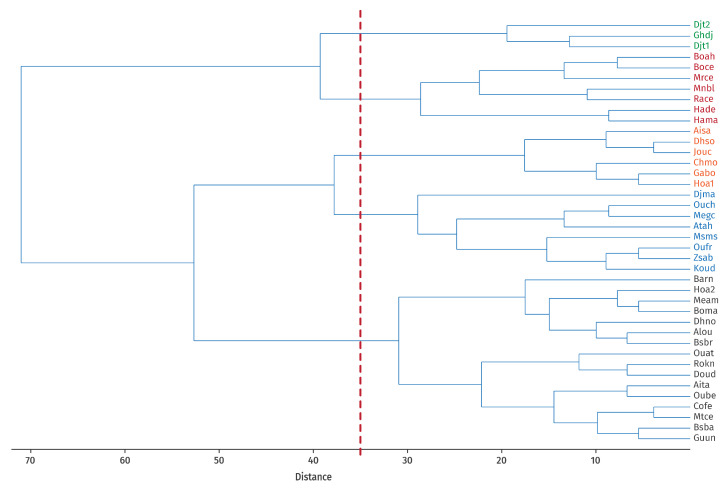
Clustering of OGUs (Operational Geographical Units) based on the habitat using Ward’s method (Cophenetic correl. 0′62) and definition of UBOs (Operational Biogeographical Units) [Binary data double standardization]. The different colours represent the distinct groups named “Operational Biogeographical Units (OBUs)”.

Based on the results of the clustering analysis (Figure 4), which shows the number of clusters obtained and validated using the cophenetic coefficient (indicating the most robust grouping according to the selected variables), the biogeographic characterization of the study area was revised according to habitat type. These newly defined biogeographic entities, termed Operational Biogeographical Units (OBUs), allowed the identification of five distinct units within Guelma Province:

**OBU1**: Included 3 stations, Djebel Taya 1 and 2 **(Djt1, Djt2)** and Ghar El Djemaa **(Ghdj)**, located at the highest elevations (1170–1260 m). Vegetation is characterized by open scrub formations dominated by *Crataegus azarolus*, with variable humidity conditions.

**OBU2**: Comprised 7 stations, Bouaicha Ahmed **(Boah)**, Boubguira cemetery **(Boce),** Merdes cemetery **(Mrce),** Menzel Bougataya Laid **(Mnbl)**, Hammam Debagh **(Hade)**, El Rahma cemetery **(Race)**, and Hammam Maskhoutine **(Hama)**, situated at medium altitudes (390–420 m). Most of these correspond to fenced cemeteries.

**OBU3**: Included 6 stations, Ain Safra **(Aisa)**, Dahouara source **(Dhso)**, Jouamaa cemetery **(Jouc)**, Chabi Mohamed **(Chmo)**, GalaatBousbaa **(Gabo)**, and Hammam Ouled Ali 1 **(Hoa1)**, located at medium altitudes. These sites consist of open scrub vegetation dominated by *Olea europaea* and *Pistacia lentiscus* L., with vegetation cover degraded by human activities such as overgrazing and agriculture.

**OBU4**: Comprised 8 stations, Madjen Barbit **(Djma)**, Oued Cheham (**Ouch**), El Megfel cemetery **(Megc)**, Ain Tahmamin **(Atah)**, Massmassa **(Msms)**, Oued Fragha **(Oufr)**, Zaouïa Sidi Abdelmalek **(Zsab)**, and El Koudia **(Koud)**. These represent semi-open preforest formations of tall scrubland with *Quercus suber*.

**OBU5**: The largest group included 16 stations, Ain Taya **(Aita)**, Ain Louza **(Alou)**, El Barnous **(Barn)**, Bouaati Mahmoud **(Boma)**, Besbessa **(Bsba)**, Bordj Sabath Road **(Bsbr)**, Colonial farm **(Cofe)**, North Dahouara **(Dhno)**, Doudou **(Doud)**, University of Guelma **(Guun)**, Hammam Ouled Ali 2 **(Hoa2)**, Medjez Amar **(Meam)**, El Metaymer cemetery **(Mtce)**, Oued d’Ain Taya **(Ouat)**, Ouled Bechih **(Oube)**, and Roknia **(Rokn)**. These are found at low to medium altitudes and are dominated by semi-open scrublands co-dominated by *Olea europaea* and *Pistacia lentiscus*, with clearings and heavily grazed mountain pastures.

### 3.5. Conditional Value (CV) for Each Taxon

**Figure 5:**
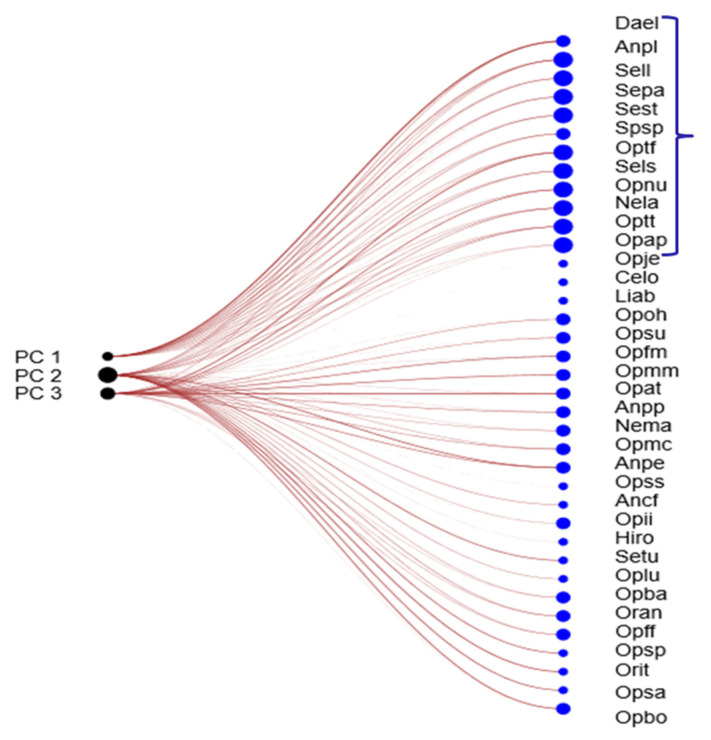
Network plot between PCs (1, 2 and 3) and taxa, through Bray–Curtis similarity index (Algorithm Bipartite linear). The most decisive group of taxa based on the weight in these three components is indicated by a bracket.

The results of the Principal Component Analysis (PCA) for components 1, 2, and 3, and their contributions to the overall set of studied taxa, are presented in Figure 5. This ordered classification reflects the degree of influence of each taxon in the characterization and clustering of the different sites, as shown in Figure 4, based on their ecological characteristics.

**Figure 6:**
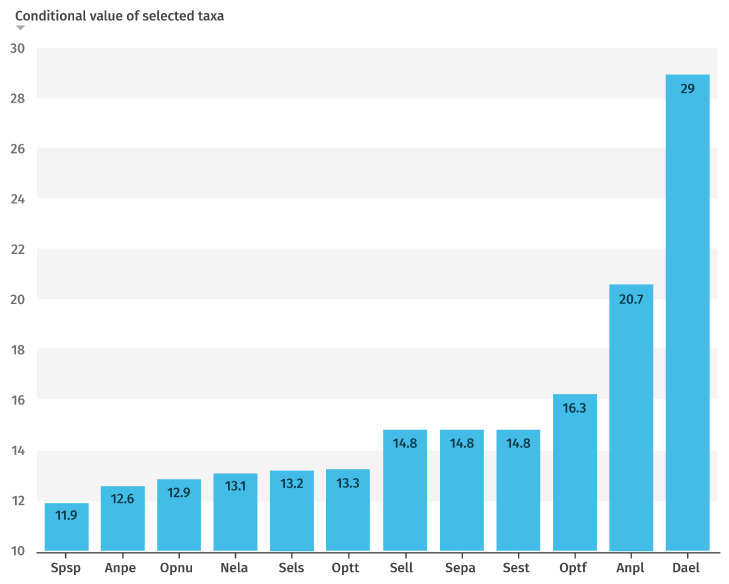
Graphical representation of the CV index values for the 12 most conditioning taxa.

Among these, the twelve most influential taxa, those with the highest values of the coefficient of variation (CV) index, are highlighted in Figure 6. Notably, *Dactylorhiza elata* exhibited the greatest weight, indicating a strong association with swamp habitats, wet meadows, stream margins, and peat bogs. This taxon is restricted to three areas where populations have declined in recent years. It occurs exclusively on peaty soils, which were previously more widespread, but are now increasingly affected by intensive agricultural and livestock activities.

### 3.6. Definition of the Eco-Chorotypes

**Figure 7:**
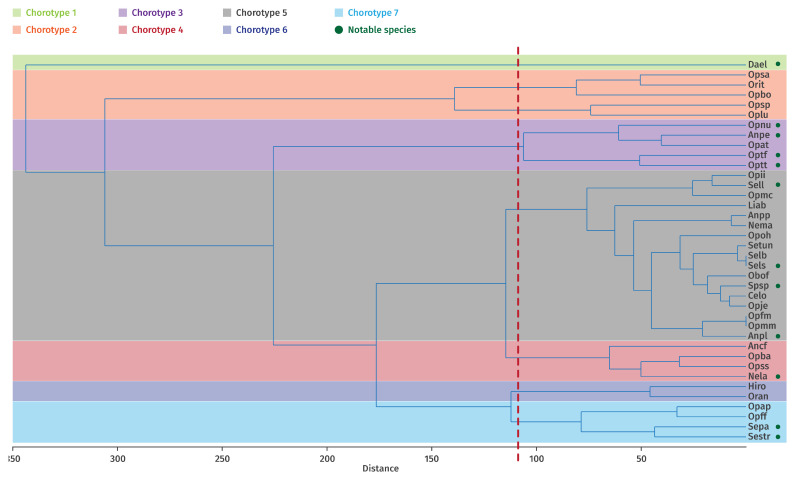
Clustering of taxa into the different identified groups using Ward’s method (cophenetic correlation 0.84) and definition of eco-corotypes (green points indicate the determining taxa according to the CV index).

The cluster analysis (Figure 7) identified seven eco-chorotypes, defined as groups of taxa sharing similar environmental requirements in relation to the ecological variables considered. The presence of key diagnostic taxa is indicated by green dots.

**Eco-chorotype** **1:** Comprises a single taxon, *Dactylorhiza elata* (**Dae**l), restricted to marshes, wetlands, stream banks, and peat bogs in open, sunny areas.
**Eco-chorotype** **2:** Includes 5 taxa, *Ophrys bombyliflora* (**Opbo**), *O. lutea* (**Oplu**), *O. scolopax* subsp. *apiformis* (**Opsa**), *O. speculum* subsp. *speculum* (**Opsp**) and *Orchis italica* (**Orit**). These are heliophilous species typically found in dry grasslands, often derived from degraded olive-mastic scrublands.
**Eco-chorotype** **3:** Formed by *Anacamptis papilionaceae* subsp. *expansa* (**Anpe**), *Ophrys atlántica* (**Opat**), *Ophrys numida* (**Opnu**), *O. tenthredinifera* subsp. *ficalhoana* (**Optf**) and *O. tenthredinifera* subsp. *tenthredinifera* (**Optt**). Located at high altitudes on loamy, calcareous, or siliceous–clay substrates.
**Eco-chorotype** **4:** Comprises *Anacamptis coriophora* subsp. *fragrans* (Synonym of *Anacamptis fragrans* -**Ancf**-), *Neotinea lactea* (**Nela**), *Ophrys battandieri* (**Opba**) and *O. scolopax* subsp. *scolopax* (**Opss**), occurring at medium altitudes on north-facing slopes with calcareous substrates.
**Eco-chorotype** **5:** High-elevation forest orchid assemblage including *Androrchis pauciflora* subsp. *laeta* (Synonym of *Orchis laeta* -**Anpl**-), *A. patens* subsp. *patens* (Synonym of *Orchis patens* -**Anpp**-), *Cephalanthera longifolia* (**Celo**), *Limodorum abortivum* (**Liab**), *Neotinea maculata* (**Nema**), *Ophrys fusca* subsp. *maghrebiaca* (**Opfm**), *Ophrys iricolor* subsp. *iricolor* [**Opii**], *O. marmorata* subsp. *marmorata* (**Opmm**), *O. marmorata* subsp. *caesiella* (Synonym of *Ophrys fusca* subsp. *bilunulata* -**Opmc**-), *O. omegaifera* (**Opoh**), *Serapias lingua* (**Sell**), *S. lingua* subsp. *stenopetala* (**Sels**), *Serapias lingua* subsp. *tunetana* (Synonym of *Serapias strictiflora* -**Setu**-), *Spiranthes spiralis* (**Spsp**) and *Ophrys × joannae* (**Opje**).
**Eco-chorotype** **6:** Comprises orchids found at medium altitudes on northern slopes with alkaline soils and early flowering phenology, including *Himantoglossum robertianum* (**Hiro**) and *Orchis anthropophora* (**Oran**).
**Eco-chorotype** **7:** Formed by 4 taxa, *Ophrys apifera* (**Opap**), *O. fusca* subsp. *fusca* (**Opff**)*, Serapias parviflora* (**Sepa**) and *S. strictiflora* (**Sest**), inhabiting moderately humid, open meadows, primarily on loamy-calcareous or sandy-clay substrates. This group appears to be largely independent of altitude.

Overall, this classification broadly supports the patterns identified through the Outlying Mean Index (OMI) analysis.

**Figure 8:**
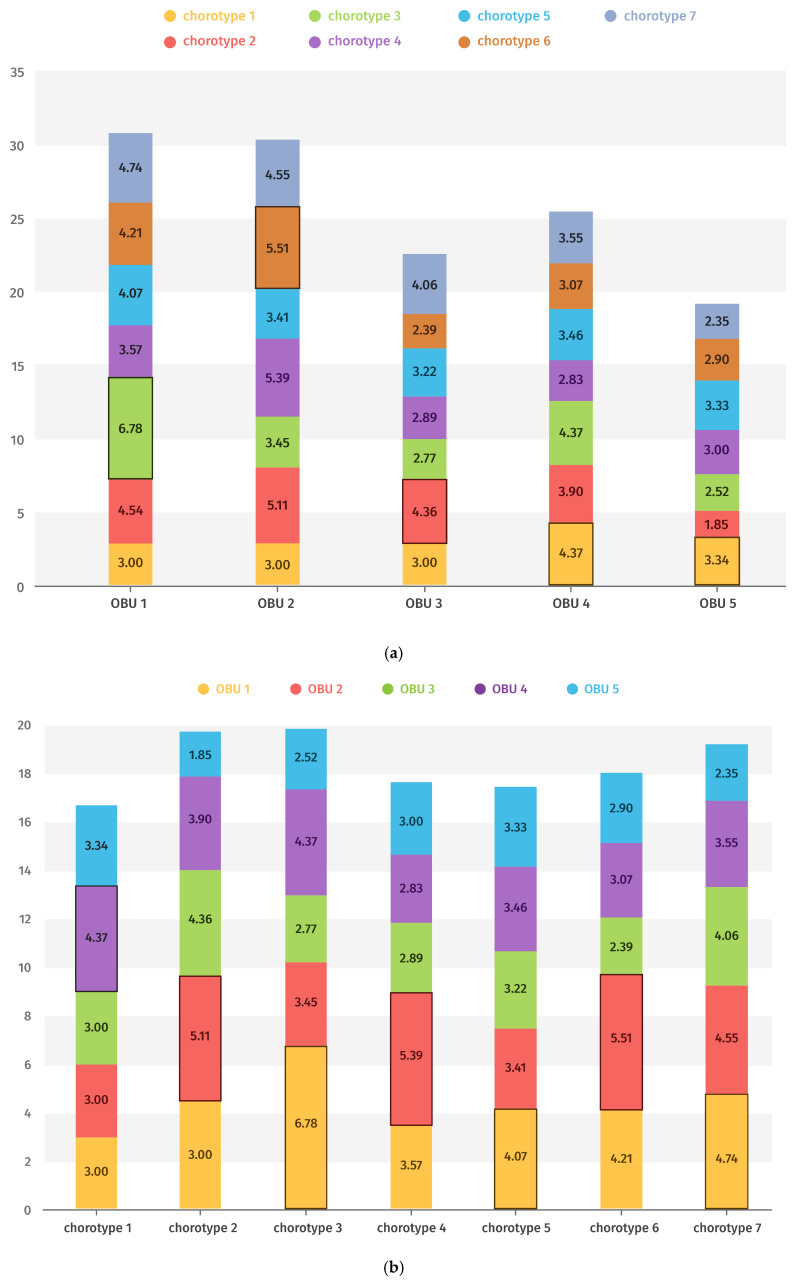
(**a**) Mean value (Axis Y) of representation in each chorotype of the different OBUs (Axis X). The most significant values are highlighted. (**b**) Mean value (Axis Y) of representation of the different chorotypes in each OBU (Axis X). The most significant values are highlighted.

The mean values of representation for each chorotype in each OBU and in each OBU of each chorotype are shown in Figure 8a,b, highlighting in each case those with the highest rank.

Figure 8a shows that Eco-chorotype 1 is the most representative of OBU 4, corresponding to scrubland areas within cork oak (*Quercus suber*) woodlands. Eco-chorotypes 2, 4, and 6 are primarily associated with OBU 2 (fenced cemeteries), while Eco-chorotypes 3, 5, and 7 are most representative of OBU 1, which includes the highest-altitude stations.

Conversely, Figure 8b indicates that the dominant eco-chorotype varies within each OBU. In OBU 1, taxa mainly belong to Eco-chorotype 3, corresponding to high-altitude species on clayey substrates; in OBU 2, most taxa are classified as Eco-chorotype 6, typical of medium-altitude environments; in OBU 3, taxa are predominantly Eco-chorotype 2, composed of heliophilous species from dry grasslands; and in OBUs 4 and 5, taxa are primarily Eco-chorotype 1, dominated by *Dactylorhiza elata*.

### 3.7. Selection of Indicator Taxa for Ecological Variables

After performing Linear r-Pearson correlations between the 37 taxa and the 9 environmental variables, Table 1 shows those that exhibited at least one correlation greater than 90%. In this way, 18 taxa were found to be directly dependent on one of these environmental variables, and these have been considered as indicators. These cases are shown in bold.

Table 1 also shows how some variables have a greater influence than others on the indicator taxa, such as tree cover or the degree of grazing-fire, compared to slope, which is not related to any of these taxa. Thus, for example, it can be observed that some species are conditioned (correlated) solely by a single variable (for instance, *Ophrys apifera*—Opap—which depends on grazing and fire, or *Neotinea maculata*—Nema—which is correlated exclusively with tree cover), whereas others depend on more than one variable. This is the case of *Ophrys tenthredinifera* subsp. *tenthredinifera* (Optt), which is strongly conditioned by exposure to sunlight, or *Serapias strictiflora* (Sest), which depends on both competition with other orchid species and tree cover (Table 2).

Among the taxa most influenced by each of the variables, those with the highest correlation are highlighted in bold, indicating a stronger dependence on that variable. Table 2 also shows that variables related to tree cover, grazing, and fire are the most decisive in determining the distribution and abundance of certain taxa.

### 3.8. Relationship Between Environmental Variables, OBUs, and Eco-Chorotypes

The results of the correlation analysis (Linear r-Pearson) between OBUs and ecological variables are shown in Table 3, where the significant are highlighted in bold on a grey background, and the most significant correlations (between 80 and 90%) for the other OBUs are shown in bold italics.

Thus, it can be observed that the groups formed in OBU 1, OBU 4, and OBU 5 are directly related to altitude, substrate, and slope, that is, to physico-chemical variables, whereas OBU 3, in addition to physical variables, also shows a close relationship with grass cover. This indicates that this group contains large open areas covered, to a greater or lesser extent, by herbaceous species.

The results of correlations (Linear r-Pearson) between Eco-chorotypes (E-Ch) and ecological variables are shown in Table 4, where significant correlations are highlighted in bold, and the most significant correlations (between 80–90%) for the other chorotypes are shown in bold italics.

In this case, it can be seen that substrate type is the variable that most strongly influences the formation of these plant groups, followed by grazing, fire, and agriculture, all of which are anthropogenic in nature. This highlights that human activities may favor the development of certain orchid taxa.

Correlation values between E-Ch and OBUs are shown in Table 5, highlighting in bold those values that are significant for each cross type.

Considering the strongest association of each eco-chorotype with the OBUs, Eco-chorotype 1 and 2, are closely linked to OBU 3 which comprises taxa typical of sunny, dry habitats, often in highly anthropized areas. Eco-chorotype 3 also preferentially correlates with OBU 1, consistent with its constituent taxa’s adaptation to high-altitude environments.

Eco-chorotype 4 is primarily associated with OBUs 2 and 3, corresponding to fenced (cemetery) and disturbed areas mainly affected by grazing. Eco-chorotype 5, the most taxa-rich group, includes forest species and correlates directly with OBU 4, composed of cork oak forests, as does Eco-chorotype 6. Finally, Eco-chorotype 7, consisting of taxa typical of wet grasslands, is associated with OBU 5, where grasslands constitute a major component of the habitat.

## 4. Discussion

### 4.1. Orchidological Potential of the Studied Region

The orchid flora of Guelma Province comprises 37 taxa, including 16 species, 19 subspecies, and 2 hybrids, representing 57.81% of all orchids recorded in Algeria [11]. This flora is dominated by the genus *Ophrys*, the most diverse orchid genus in North Africa [12,13].

Within the Numidian sub-sector of northeastern Algeria, which includes five provinces, including Guelma, de Bélair et al. (2005) [26] reported 34 orchid taxa, three fewer than identified in the present study. Compared to Souk-Ahras (approximately 2500 km^2^), Guelma harbors ten additional taxa. The number of taxa recorded here also exceeds that reported by Bougaham et al. (2015) [56] in the Kabylia of the Babors, who recorded 27 taxa in an area ten times smaller. Similarly, Hamel and Meddad-Hamza (2016) [27] reported 20 taxa for the Edough Peninsula, and Hamel et al. (2017) [28] recorded 18 taxa in Skikda Province. Other regions, such as the Aurès, have yielded fewer taxa, with Beghami et al. (2015) [57] reporting only nine taxa.

In terms of distribution, *Orchis laeta*, an Algerian–Tunisian endemic [2], was observed within a restricted area (100–5000 km^2^). The endemic *Serapias lingua* subsp. *stenopetala* occupies an even smaller range, less than 100 km^2^ [37,56].

Four taxa are listed in the IUCN Red List [36] with different statuses: Near Threatened (NT) for *Orchis laeta* and *Dactylorhiza elata*; Endangered (EN) for *Ophrys atlantica*; and Critically Endangered (CR) for Serapias lingua subsp. *stenopetala*. Moreover, six taxa are included in the Algerian list of protected non-cultivated plant species, which comprises 449 taxa [50].

Regarding orchids of patrimonial value (regional endemics, nationally rare, or globally threatened), three are associated with wet meadows, ponds, and springs (*Dactylorhiza elata*, *Serapias lingua* subsp. *stenopetala*, *S. tunetana*), while the remaining taxa are linked to open Mediterranean shrublands at low to medium altitudes. The Djebel Taya station, which hosts *Dactylorhiza* specimens alongside *Serapias*, thus has particular patrimonial importance. However, these taxa face numerous threats, including trampling and collection, as this region has experienced a growing influx of visitors in recent years [31].

Due to the lack of systematic studies, most endemic orchids in the study area and across Algeria have not yet been formally assessed according to IUCN criteria (only hygrophytic and amphiphytic taxa have been evaluated) [38]. Based on the selection criteria used in the IUCN Red List (version 3.1, 2001) [50] and the results of a national preliminary assessment, five orchid taxa have been assigned provisional statuses [26,29,31]: one subspecies is Endangered (EN) (*Orchis patens*), three are Near Threatened (NT) (*Ophrys battandieri*, *O. fusca* subsp. *maghrebiaca*, *Serapias lingua* subsp. *lingua*), and one is Data Deficient (DD) (*Serapias lingua* subsp. *tunetana*).

Currently, these orchids face significant threats from habitat loss, primarily driven by agriculture, overgrazing, and wildfires [31]. The high regeneration rate observed in heliophilous orchids may reflect the impact of intensified agro-pastoral activities, which act as major disturbances, particularly through the conversion of old forest ecosystems into grazing areas. The persistence of orchid populations after fire events provides valuable information on the soil depth reached by lethal temperatures [58] and can also serve as an indicator of post-fire mycorrhizal fungal activity [59,60].

Several taxa remain legally unprotected due to historical taxonomic changes. Taxa previously classified at the infraspecific level in the reference flora [35] were not included in the executive decrees of 23 November 1993 and 4 January 2012. Nevertheless, such gaps should not hinder the conservation of local biodiversity [58]. Conservation priorities should therefore be guided by endemism and rarity: (i) immediate protection of taxa that are both endemic and rare, along with their habitats; (ii) subsequent measures for rare but non-endemic taxa; and (iii) eventual inclusion of less rare endemic taxa in broader management and conservation plans. Comparison of the present results with those of de Bélair et al. (2005) [26] reveals the presence of 20 newly recorded taxa. In addition, eight orchid taxa poorly known from the study area [35] have been confirmed, highlighting both a historical gap in floristic surveys and the need for updated regional inventories:

▪ ***Orchis patens***. This taxon appears to be rare in the study region, as it has only been observed at a single site (Ghar El Djemaa), with approximately twenty individuals. It is scattered across the Kabylias–Numidia–Kroumiria mini-hotspot [30,32] and in the coastal subsectors of Algiers (“A_1_”) and Oran (“O_1_”) [3]. In the Hills of Constantine sector, the species has been previously reported in Souk Ahras Province [26,29]. Its occurrence in the Guelma Province is noteworthy, as this taxon is endemic to Algeria, Tunisia, and northwestern Italy [35]. Nevertheless, it remains extremely rare, and its typical habitats—open woodlands and scrublands—are severely threatened by grazing, agriculture, trampling, and forest fires [58].
▪ ***Cephalanthera longifolia*.** A single individual of this taxon was recorded in the Ouled Bechih area, within a mixed oak forest. According to Quézel & Santa (1962) [35], it was formerly considered fairly common in the Tell region, which is no longer the case based on recent surveys conducted in the study area and neighbouring regions such as Souk Ahras, where the localities cited by de Bélair et al. (2005) [26] are no longer extant. This semi-forest orchid is threatened by the degradation and disappearance of forest undergrowth, mainly due to overgrazing, which removes the organic soil layer and the shrub stratum, thereby endangering forest regeneration in the medium term [26]. The species therefore deserves special conservation attention.
▪ ***Ophrys fusca* subsp. *bilunulata***. This taxon has previously been recorded in the Hills of Constantine (C_1_) by Boukehili et al. (2018) [29] in Souk Ahras Province. It is newly reported here for Guelma Province, where it occurs in large numbers within wild olive–mastic type scrub.
▪ ***Ophrys omegaifera* subsp. *hayekii***. This taxon was first described in Algeria, in Small Kabylia (March, 2007) by Rebbas & Véla (2008) [61] under the name Ophrys mirabilis P. Geniez & F. Melki. It was subsequently recorded in El-Hodna (“Hd”) by Bounar et al. (2012) [62], in M’Sila (“H_2_”) by Rebbas et al. (2017) [63], in the Kabylias–Numidia–Kroumiria sectors (“K_1_, K_2_-K_3_”) by Martin et al. (2020) [11], in the Djurdjura by Kreutz et al. (2013, 2014) [64,65], in Béni Foughal (Jijel) by Hadji & Rebbas (2014) [66], in Souk Ahras by Boukehili et al. (2018) [29], and recently in the Hills of Tlemcen by Babali et al. (2018) [67]. Because of its recent description, it is absent from the national floras of Algeria [58] and Italy [46]. In the present study area, it is very poorly represented, recorded only at a single site (Ghar El Djemaa, 1094 m, northern foothills of the Roknia massif), with about 30 individuals.
▪ ***Serapias lingua* subsp. *stenopetala***. Originally described as a subspecies of *S. lingua* [43], this taxon was later detailed by de Bélair & Boussouak (2002) [68]. Endemic to Algeria and Tunisia, it typically grows in wet peatlands and is often associated with *Juncus maritimus* Lam. communities [69]. In Numidia, it is considered very rare due to the scarcity of both individuals and localities [26,27,29]. It is listed by the IUCN as Critically Endangered (CR) [69].
▪ ***Serapias lingua* subsp. *tunetana***. Observations by El Mokni et al. (2012) [70] in Tunisia, Algeria, and Morocco have enabled morphological differentiation of *S. tunetana* from *S. lingua*, justifying its elevation to species rank (*S. tunetana*). Nationally, it was first recorded in sector C_1_ (Djbel El Ouahch, Hills of Constantine) and in subsector K_3_ (El Kala National Park). In Guelma Province, it was observed at a single sampling site, Madjen Barbit, in May 2024.
▪ ***Ophrys* × *joannae***. This hybrid (*O. atlantica* × *O. omegaifera* subsp. *hayekii*) has been formally recognised only in Souk Ahras Province (Eastern Algeria) [29], where it is very rare. It has also been observed in the Tlemcen and Ghar Roban mountains (Western Algeria) [71], and in Tunisia on Mount Bou-Kornine [72]. In the present study area, it is likewise rare, recorded only at Ghar El Djemaa (eight individuals) in a degraded thicket with *Erica arborea* L. and *Calicotome villosa* (Poir.) Link, in May 2017.
▪ ***Ophrys fusca* subsp. *fusca***. This taxon was observed as a single individual among stands of *Pistacia lentiscus* in the locality of Guigueba. It is generally widespread across the Mediterranean Basin [73]. However, it is not listed in the Algerian flora of Quézel & Santa (1962) [35] nor in the North African flora of Maire (1960) [43], and its presence is also doubtful in Tunisia [73]. More recent floristic reviews of North Africa [24] also regard its presence in Tunisia as uncertain. Nevertheless, its confirmed identification here, well outside its classical western Mediterranean range [44], suggests that additional populations may exist and merit targeted investigation within its potential range.

### 4.2. Orchid Diversity in the Mini-Hotspot Kabylias–Numidia–Kroumiria

In northeastern Algeria, the provinces exhibiting the highest orchid diversity are Souk Ahras, with 27 taxa (6 of which are endemic), and the Babors, also with 27 taxa but only 3 endemics (Table S5). The exceptional habitat diversity in both areas plays a decisive role in sustaining this richness [29,32]. El Kala National Park in El Tarf Province follows, containing 23 taxa, including 4 endemics *Orchis patens*, *Ophrys numida*, *Platanthera bifolia* subsp. *bifolia*, and *Serapias lingua* subsp. *stenopetala*) [30]. The Edough Peninsula harbours 20 taxa, two of which are endemic to Algeria and Tunisia (*Platanthera bifolia* subsp. *bifolia* and *Serapias lingua* subsp. *stenopetala*) [27]. In this peninsula, scrublands and xeric grasslands host all observed orchid species, occupying about one quarter of its total surface area [74].

The Skikda Province exhibits comparatively lower diversity, with 18 taxa and a single endemic species (*Dactylorhiza elata*) [28,75].

In northern Tunisia, the Kroumiria and Mogods mountains—bordering the Mediterranean and forming the eastern terminus of the Tellian Atlas, which extends across northern Algeria, also exhibit high orchid diversity [45]. A total of 23 taxa have been recorded in this region, including four endemics. Among these, five taxa are newly reported for the Mogods complex and the Medjerda Valley, including three *Ophrys* taxa that share patterns of endemism with neighboring Mediterranean regions (Algeria, Libya, and Sicily) [72].

All these taxa are either endemic to the regional hotspot Kabylias–Numidia–Kroumiria [7] (Table S5) or subendemic, with additional disjunct distributions. They therefore hold high conservation value due to both their biogeographical significance and threatened status [76].

The inventories conducted to date account for approximately 49.33% of Tunisia’s orchid flora [24], exceeding the figures reported for other northeastern regions within or adjacent to the Kabylias–Numidia–Kroumiria biodiversity hotspot [7]. In this context, the 37 taxa recorded in Guelma Province are particularly noteworthy, as this area lies within the natural distribution range of most Algerian orchids [11].

### 4.3. Orchid Ecological Requirements

Most of the orchids inventoried tend to colonize wild olive–mastic shrublands in the study region, particularly in areas with abundant grasses. These open and sparsely vegetated habitats appear to be favorable environments for orchids, both in terms of taxa richness and individual abundance [77]. Among the 40 sites surveyed, seven correspond to cemeteries, which together contain nearly two-thirds of all recorded orchids, mainly in El Rahma and Merdes cemeteries. This type of habitat is globally recognized as a refuge for diverse plant species, as demonstrated by Hadi et al. (2014) [78] in Pakistan and Nagy et al. (2025) [41] in Europe, who showed that cemeteries can serve as sanctuaries for plant taxa extinct or nearly extinct in surrounding areas. Being fenced, these sites are protected from grazing and trampling by animals and humans, providing shelter even for rare orchids such as *Ophrys fusca* subsp. *bilunulata* and *Ophrys battandieri* [26]. In addition, numerous localities are situated near roadsides, on embankments, verges, or within fragments of *Olea–Pistacia* shrubland, some adjacent to major roads, such as at the El Megfel cemetery site. This ecological plasticity acts as a form of spontaneous protection [79].

However, open habitats such as sparse, low-density shrublands do not appear to be in an optimal conservation state, despite supporting a high number of taxa and individuals. Twenty taxa recorded in this study were not listed by de Bélair et al. (2005) [26], suggesting that the present field survey was extensive and that a high turnover rate may characterize this heliophilous orchid flora. This pattern likely reflects intensified agricultural and pastoral activities, which have increasingly altered the landscape through the conversion of former forest ecosystems into pastureland. It may also indicate that these ecosystems are still in an early stage of transformation, with orchid taxa beginning to exhibit resilience and partial adaptation to recent environmental changes.

Each orchid taxon in the Guelma Province displays local ecological and microclimatic adaptations that compensate for regional constraints, enabling persistence within conditions compatible with their natural biogeography [31]. Some species, such as *Neotinea maculata* and *Ophrys fusca* subsp. *fusca*, typical Mediterranean taxa of subhumid habitats, are mainly found in cooler microclimates [45]. In contrast, others, like *Orchis anthropophora*, a Mediterranean–Atlantic taxon associated with semiarid conditions, are found in sun-exposed scrublands [80]. Several resilient taxa, well adapted to current environmental conditions, are widely present in pre-forest and shrubland formations across the region.

The forest orchid group is represented by *Cephalanthera longifolia* and *Limodorum abortivum*, both understory species described by de Bélair et al. (2005) [26] in Numidia. These orchids are threatened by the degradation or disappearance of forest habitats, such as those of Beni Salah and Djebel Taya, where cork extraction is common and contributes to the deterioration of the shrub layer in cork oak woodlands [31].

Most orchids recorded in the study area belong to grassland heliophilous groups. These grasslands, typically derived from degraded forests, are characterized by a predominance of annual taxa. Prolonged human and livestock pressure have led to the selection of unpalatable or resistant species, with therophytes playing an important role in these communities [81].

The distribution of orchid taxa varies according to environmental conditions. Most taxa occur in the northern part of Guelma Province, characterized by clayey-sandstone or marl–limestone substrates. Taxa of the genus *Ophrys* (*O. tenthredinifera* subsp. *tenthredinifera*, *O. lutea*, *O. scolopax* subsp. *apiformis*, *O. scolopax* subsp. *scolopax*, and *O. bombyliflora*) occupy medium-altitude stations, mainly on northern slopes with calcareous or siliceous–clay substrates, where woody cover is low and the herbaceous layer is dense. Similar ecological conditions were reported by El Karmoudi et al. (2025) [13] in the West Rif region of northern Morocco.

The southern part of the province, dominated by limestone substrates and low woody cover on medium to steep slopes, hosts twelve taxa, with *Orchis anthropophora* and *Anacamptis papilionacea* subsp. *expansa* being the most common. These taxa are well adapted to prevailing conditions and are widely distributed within pre-forest formations [12,16]. Notably, the results indicate that many orchids typically considered calcicolous also occur, and even thrive on non-calcareous substrates. For instance, *Ophrys numida*, *Orchis italica*, and *Himantoglossum robertianum* were observed on soils derived from non-calcareous rocks, whereas previous studies [9,82] described these taxa as strictly calcicolous.

The 37 orchids recorded occur at altitudes ranging from 165 to 1055 m. High-altitude stations (Ghar El Djemaa, Djebel Taya, Massmassa, and Madjen Barbit) contain not only the greatest number of taxa but also those of high patrimonial value. Similarly, Gerakis et al. (2025) [83] found that altitude, geological substrate, and specific habitat type are the primary factors shaping orchid distribution in northeastern Greece. Some orchids in the present study were found exclusively in high-altitude wetlands, which represent sensitive and ecologically important habitats. In these wetlands, *Serapias* spp. and *Dactylorhiza elata* were abundant, occupying shaded exposures that minimize soil desiccation and evapotranspiration due to solar radiation [77].

Overall, the habitat and ecological patterns observed for the orchids recorded in this study are broadly consistent with those reported by Quézel and Santa (1962) [35], de Bélair et al. (2005) [26], and Martin et al. (2015) [45].

### 4.4. Biogeographical and Conservation Interest

As previously noted by Boukehili et al. (2018) [29], Mediterranean elements dominate the orchid flora of Souk Ahras Province—a pattern confirmed in the present study, where Mediterranean taxa are likewise predominant. Similar trends have been reported for the orchid flora of northeastern Algeria by Hamel and Meddad-Hamza (2016) [27] and Boutabia et al. (2019) [30]. In contrast, the orchid flora of the Oranais region in northwestern Algeria is influenced by the western Mediterranean subdomain, including the Ibero-Maghrebian complex [12], whereas northeastern Algeria falls under the influence of the central Mediterranean subdomain, associated with the Tyrrhenian microplate complex [56,84].

The presence of several endemic and/or threatened taxa shared with adjacent floristic regions suggests that the study area may constitute a well-defined sub-IPA (Important Plant Area). It forms part of the El Kala 2 IPA, previously identified further north, with which it maintains complete geographical and ecological continuity [37,85].

The presence of 37 taxa in the study area highlights the exceptional richness of its orchid flora and underscores the region’s significant conservation responsibility. In this context, the implementation of protection measures is essential, particularly through the regulation of emerging non-traditional agricultural practices that may adversely affect both the orchids and their habitats.

Most of the habitats favorable to these taxa are currently in a critical state, with the exception of cemeteries, which remain protected and thus largely shielded from regional disturbances. Controlled grazing, excluding orchid sites during flowering and fruiting periods, appears to be an effective and practical conservation measure for safeguarding these rare and vulnerable taxa.

One of the objectives of this study was to update current knowledge on the diversity and ecological potential of the orchid flora. Further floristic investigations are urgently needed in Guelma Province, particularly in degraded or insufficiently explored habitats, with the aim of establishing a comprehensive orchid distribution map for the region. Such a tool, currently unavailable, will be essential for developing rigorous conservation and protection programs.

In this regard, it is necessary to undertake studies supporting the classification of the study area—or parts of the Djebel Taya, Beni Salah, and El Mezeraa forest massifs—as a National Natural Park, with the goal of ensuring biological stability and safeguarding its natural heritage [31,59]. This designation is justified by the substantial number of endemic, rare, threatened, and protected taxa, as well as the habitats they occupy.

It is equally important to promote conservation policies that consider the socioeconomic needs of local communities. Finally, the competent authorities must be encouraged to enforce legal protection measures to preserve this vulnerable plant group, whose biological and heritage value is clearly established. As in other regions of Algeria, several ecosystems and natural areas in Guelma remain understudied, yet they may hold significant biological and ecological importance.

## Figures and Tables

**Table 1:** Linear r-Pearson correlation between ecological variables and indicator taxa. The strongest correlations are indicated in bold.

	Alt	Subs	Exp	Slop	Orch-Surf	Tree-Cover	Grass-Cover	Pasture-Fire	Agric
**Nema**	0.45	0.83	0.74	0.87	0.14	**0.93**	0.53	0.59	0.67
**Opap**	0.17	0.1	0.52	0.09	0.36	0.45	0.51	**0.91**	0.43
**Opba**	0.4	**0.93**	0.77	0.35	0.01	0.68	0.04	0.51	0.71
**Opfm**	0.45	0.83	0.86	0.37	0.62	0.39	**0.91**	0.15	0.14
**Opii**	0.16	**0.93**	0.74	0.35	0.74	0.68	0.11	0.22	0.43
**Opmc**	0.77	0.83	0.49	0.87	0.14	**0.93**	0.1	0.42	0.67
**Opmm**	0.24	0.48	0.23	0.53	0.49	0.55	0.55	**0.95**	0.71
**Opss**	0.18	0.55	0.2	0.53	0.67	0.29	0.29	**0.91**	0.52
**Opsp**	0.49	0.49	0.15	0.26	0.57	0.59	0.25	0.36	0.71
**Optf**	0	0.14	0.34	0.26	0.77	**0.91**	0.22	0.36	**0.96**
**Optt**	0.34	0.11	**0.91**	0.32	0.34	**0.9**	0	0.11	0.35
**Oran**	**0.99**	0.38	0.77	0.77	0.01	0.68	0.04	0.51	0.71
**Orit**	0.52	0.45	0.89	0.08	0.6	**0.98**	0.02	**0.98**	0.02
**Sell**	0.09	0.55	**0.99**	0.26	0.04	0.19	0	0.16	0.35
**Sels**	0.07	0.48	0.23	0.03	**0.99**	0.03	0.1	0.57	0.33
**Sepa**	0.29	0.34	0.46	0.71	0.25	**0.93**	0.16	0.75	0.05
**Setu**	0.07	0.48	0.23	0.03	**0.99**	0.03	0.1	0.57	0.33
**Sest**	0.57	0.48	0.29	0.31	**0.96**	**0.98**	0.4	0.8	0.08

**Table 2:** Taxa selected for each variable.

**Altitude**	Oran						
**Substrate**	Opba	Opii					
**Exposure**	Optt	**Sell**					
**Slope**	-						
**Orchidological surface**	**Sels**	Setu	Sest				
**Tree cover rate**	Nema	Opmc	Optf	Optt	**Orit**	Sepa	**Sest**
**Grass cover rate**	Opfm						
**Pasture fire**	**Opap**	Opmm	**Opss**	Orit			
**Agricultural activities**	**Optf**						

**Table 3:** Linear r-Pearson correlation between OBUs and ecological variables.

	Alt	Subs	Exp	Slop	Orch-Surf	Tree-Cover	Grass-Cover	Pasture-Fire	Agric
**OBU 1**	***0.84***	***0.81***	0.30	***0.85***	0.06	0.54	0.04	0.79	0.18
**OBU 2**	0.00	0.07	0.47	0.45	0.08	0.47	0.01	0.02	0.10
**OBU 3**	0.20	**0.96**	0.01	***0.82***	0.67	0.52	**0.94**	0.07	0.35
**OBU 4**	0.17	0.01	**0.96**	0.01	0.22	0.44	0.15	0.19	0.52
**OBU 5**	0.03	***0.85***	0.70	0.32	0.23	0.07	0.04	0.03	0.44

**Table 4:** Linear r-Pearson correlation between Eco-chorotypes and ecological variables.

	Alt	Subs	Exp	Slop	Orch-Surf	Tree-Cover	Grass-Cover	Pasture-Fire	Agric
E-Ch 01	0.00	0.21	0.39	0.26	0.22	0.61	0.09	0.16	***0.89***
E-Ch 02	0.46	**0.90**	0.07	0.09	0.03	0.14	0.00	0.77	0.13
E-Ch 03	0.00	0.23	0.69	**0.97**	0.03	0.39	0.01	0.77	**0.97**
E-Ch 04	0.44	**1.00**	0.25	0.67	0.02	0.25	0.05	***0.84***	0.34
E-Ch 05	0.00	0.67	0.23	0.09	0.01	0.21	0.11	***0.86***	**0.99**
E-Ch 06	0.56	**0.99**	0.61	0.33	0.03	0.68	0.04	0.13	0.53
E-Ch 07	0.32	0.14	0.66	0.34	0.25	**0.93**	0.08	0.87	0.04

**Table 5:** Correlation values between Eco-chorotypes (E-Ch) and OBUs.

	E-Ch 01	E-Ch 02	E-Ch 03	E-Ch 04	E-Ch 05	E-Ch 06	E-Ch 07
**OBU 1**	0.41	0.04	**0.61**	0.00	**0.79**	0.24	0.39
**OBU 2**	0.29	0.24	0.20	**0.80**	0.48	0.45	0.18
**OBU 3**	**0.67**	**0.83**	0.38	**0.96**	0.56	**0.95**	0.12
**OBU 4**	0.13	0.28	0.42	0.25	**0.83**	0.63	0.37
**OBU 5**	0.50	**0.59**	0.51	0.36	0.42	0.06	**0.48**

## Data Availability

The original contributions presented in the study are included in the Article/Supplementary Materials. Further inquiries can be directed to the corresponding author.

## Supplementary Materials

The following supporting information can be downloaded at https://www.mdpi.com/article/10.3390/plants14243833/s1. Table S1: Orchid diversity and values of the variables considered at each sampling station; Table S2: Biogeographical and heritage value of orchids in the Guelma province; Table S3: Orchid diversity in the mini-hotspots (Kabylias–Numidia–Kroumiria); Table S4: Sampling stations at Guelma province; Table S5: Environmental characteristics measured.

## Appendix A

Floristic catalogue and classification according to Chase et al. (2015) [86] and Benito-Ayuso (2017) [87]. Acronyms are included for each taxon.

Fam. *Orchidaceae*
  Subfam. Epidendroideae
    Tribe *Neottieae*
      Subtribe Limodorinae
        - *Cephalanthera longifolia* (L.) Fritsch. [Celo]
        - *Limodorum abortivum* (L.) Sw. [Liab]
  Subfam. Orchidoideae
    Tribe Cranichideae
      Subtribe Spiranthinae
        - *Spiranthes spiralis* (L.) Chevall. [Spsp]
    Tribe Orchideae
      Subtribe Orchidinae
        - *Anacamptis coriophora* (L.) R.M. Bateman, Pridgeon & M.W. Chase subsp. *fragrans* (Pollini) R.M. Bateman, Pridgeon & M.W. Chase (Synonym of *Anacamptis fragrans* (Pollini) R.M. Bateman) [Ancf]
        - *Anacamptis papilionacea* R.M. Bateman, Pridgeon & M.W. Chase subsp. *expansa* (Ten.) Amard. & Dusak [Anpe]
        - *Androrchis patens* (Desf.) D. Tyteca & E. Klein subsp. *patens* (Synonym of *Orchis patens* Desf.) [Anpp]
        - *Androrchis pauciflora* (Ten.) D. Tyteca & E. Klein subsp. *laeta* (Steinh.) Véla, Rebbas & R. Martin Comb. Nov. (Synonym of *Orchis laeta* Steinh). [Anpl]
        - *Dactylorhiza elata* (Poir.) Soó [Dael]
        - *Himantoglossum robertianum* (Loisel.) P. Delforge [Hiro]
        - *Neotinea lactea* (Poir.) R. M. Bateman, A. M. Pridgeon & M. W. Chase [Nela]
        - *Neotinea maculata* (Desf.) Stearn [Nema]
        - *Ophrys apifera* Huds. [Opap]
        - *Ophrys atlantica* Munby [Opat]
        - *Ophrys battandieri* E.G. Camus, P. Bergon & A. Camus [Opba]
        - *Ophrys bombyliflora* Link [Opbo]
        - *Ophrys fusca* Link subsp. *fusca* [Opff]
        - *Ophrys fusca* Link subsp. *maghrebiaca* Kreutz et al. [64,65] [Opfm]
        - *Ophrys iricolor* Desf. subsp. *iricolor* [Opii]
        - *Ophrys lutea* Cav. subsp. *lutea* [Oplu]
        - *Ophrys marmorata* G.Foelsche & W.Foelsche subsp. *caesiella* (P. Delforge) E. Véla & R. Martin (Synonym of *Ophrys fusca* subsp. *bilunulata* (Risso) Kreutz) [Opmc]
        - *Ophrys marmorata* G. Foelsche & W. Foelsche subsp. *marmorata* [Opmm]
        - *Ophrys numida* Devillers-Terschuren & Devillers (Synonym of *Ophrys murbeckii* H.Fleischm.) [Opnu]
        - *Ophrys omegaifera* H. Fleischm. subsp. *hayekii* (H. Fleischm. & Soó) Kreutz [Opoh]
        - *Ophrys scolopax* Cav. subsp. *apiformis* (Desf.) Maire & Weiller [Opsa]
        - *Ophrys scolopax* Cav. subsp. *scolopax* [Opss]
        - *Ophrys speculum* Link subsp. *speculum* [Opsp]
        - *Ophrys tenthredinifera* Willd. subsp. *ficalhoana* (J.A. Guim.) M.R. Lowe & D. Tyteca [Optf]
        - *Ophrys tenthredinifera* Willd. subsp. *tenthredinifera* [Optt]
        - *Ophrys* x *joannae* Maire [Opje]
        - *Ophrys* x *sommieri* Sommier ex E.G. Camus [Opsu]
        - *Orchis anthropophora* (L.) All. [Oran]
        - *Orchis italica* Poir. [Orit]
        - *Serapias lingua* L. subsp. *lingua* [Sell]
        - *Serapias lingua* L. subsp. *stenopetala* (Maire & T. Stephenson) Maire & Weiller [Sels]
        - *Serapias lingua* L. subsp. *tunetana* B. Baumann & H. Baumann (Synonym of *Serapias strictiflora* Welw. ex Da Veiga) [Setu]
        - *Serapias parviflora* Parl. [Sepa]
        - *Serapias strictiflora* Welw. ex Da Veiga [Sest]
