# Supplementary material for: Orchid Flora of the Guelma Region (North-Eastern Algeria), a Little-Known Group for Algerian Flora
Source: Plants (Basel). 2025 Dec 16;14(24):3833. doi: 10.3390/plants14243833 (PMC12736946; doi:10.3390/plants14243833)
Supplement: Supplementary file 1 [file plants-14-03833-s001.zip › plants-4002307-supplementary.pdf]

Supplementary material

Table S1. Sampling stations at Guelma province

Code	Station	GPS	Average Alt. (masl)	Type of vegetation	Orchidological surface
Alou	Ain Louza	36°28'45"N ; 7°13'24"E	615	High scrub with wild olive and mastic trees	70
Aisa	Ain Safra	36°21'45"N ;7°40'43"	647	Medium scrub with wild olive and mastic trees	50
Atah	Ain Tahmamin	36°24'53"N ;7°48'26"E	333	Cork oak grove dominated by *Quercus suber* L.	50
Aita	Ain Taya	36°30'27"N ; 7°06'45"E	1004	High scrub with *Crateagus azarolus* L.	200
Bsba	Besbessa	36°19'32"N ;7°44'03"E	721	High scrub with wild olive and mastic trees	45
Bsbr	Bordj Sabath Road	36°24'58"N ;7°03'43"E	678	High scrub with wild olive and mastic trees	250
Boma	Bouaati Mahmoud	36°38'44"N ;7°19'39"E	95	Medium scrub with wild olive and mastic trees	50
Boah	Bouaicha Ahmed	36°35'21"N ;7°30'33"E	353	Medium scrub with wild olive and mastic trees	650
Boce	Boubguira cemetery	36°26'35"N ; 7°50'55"E	294	Low scrub with wild olive and mastic trees	900
Chmo	Chabi Mohamed	36°34'28"N ; 7°30'02"E	571	Medium scrub with wild olive and mastic trees	750
Cofe	Colonial farm	36°24'42"N ; 7°25'52"E	475	Medium scrub with wild olive and mastic trees	60
Dhso	Dahouara source	36°21'17"N ; 7°44'26"E	415	Medium scrub with wild olive and mastic trees	100
Djt1	Djebel Taya 1	36°30'38"N ; 7°05'43"E	1037	High scrub with *Crateagus azarolus* L.	490
Djt2	Djebel Taya 2	36°30'32"N ; 7°05'47"E	1043	High scrub with *Crateagus azarolus* L.	500
Doud	Doudou	36°17'52"N ; 7°35'07"E	930	Low scrub with *Crateagus azarolus* L.	300
Barn	El Barnous	36°20'20"N ; 7°38'10"E	562	Medium scrub with *Olea europaea* L. and *Quercus suber* L.	200
Koud	El Koudia	36°30'25"N ; 7°08'51"E	919	Scrub with *Quercus suber* L.	400
Megc	El Megfel cemetery	36°23'00"N ; 7°48'22"E	502	Cork oak grove dominated by *Quercus suber* L.	350
Mtce	El Metaymer cemetery	36°21'14"N ; 7°35'56"E	540	Medium scrub with wild olive and mastic trees	300
Race	El Rahma cemetery	36°26'19"N ; 7°46'21"E	181	Low scrub with wild olive and mastic trees	850
Gabo	GalaatBousbaa	36°31'55"N ; 7°29'02"E	419	Low scrub with wild olive and mastic trees	80
Ghdj	Ghar El Djemaa	36°30'30"N ; 7°08'19"E	957	High scrub with *Crateagus azarolus* L.	450
Hade	Hammam Debagh	36°28'13"N ; 7°15'40"E	310	Medium scrub with wild olive and mastic trees	700
Hama	Hammam Maskhoutine	36°26'23"N ; 7°17'56"E	295	Medium scrub with wild olive and mastic trees	150
Hoa1	Hammam Ouled Ali 1	36°33'58"N ; 7°21'59"E	236	Medium scrub with wild olive and mastic trees	50
Hoa2	Hammam Ouled Ali 2	36°34'38"N ; 7°23'04"E	233	Medium scrub with wild olive and mastic trees	40
Jouc	Jouamaa cemetery	36°23'03"N ; 7°40'31"E	559	Low scrub with *Crateagus azarolus* L.	100
Dima	Madjen Barbit	36°22'09"N ; 7°23'47"E	1259	Wet grassland with *Juncus anceps* Laharpe and *Typha domingensis* Pers.	300
Msms	Massmassa	36°21'54"N ; 7°24'53"E	1003	Wet grassland with *Juncus anceps* Laharpe and *Typha domingensis* Pers.	1050
Meam	Medjez Amar	36°26'29"N ; 7°18'40"E	249	Low scrub with wild olive and mastic trees	70

Mnbl	Menzel Bougataya Laid	36°22'31"N ; 7°46'48"E	542	Medium scrub with wild olive and mastic trees	100
Mrce	Merdes cemetery	36°27'07"N ; 7°45'57"E	242	Low scrub with wild olive and mastic trees	450
Dhno	North Dahouara	36°20'53"N ; 7°41'42"E	321	Medium scrub with wild olive and mastic trees	150
Ouch	Oued Cheham	36°22'26"N ; 7°48'33"E	534	Medium scrub with wild olive and mastic trees	45
Ouat	Oued d'Ain Taya	36°30'10"N ; 7°06'30"E	964	Riparian forest with *Juncus articulatus* L.	220
Oufr	Oued Fragha	36°34'14"N ; 7°40'45"E	165	Low scrub with wild olive and mastic trees	45
Oube	Ouled Bechih	36°24'18"N ; 7°51'47"E	819	Cork oak grove dominated by *Quercus suber* L.	80
Rokn	Roknia	36°35'27"N ; 7°13'16"E	143	Low scrub with wild olive and mastic trees	70
Guun	University of Guelma	36°26'46"N ; 7°24'58"E	417	Medium scrub with wild olive and mastic trees	50
Zsab	Zaouïa Sidi Abdelmalek	36°23'04"N ; 7°25'34"E	692	Low scrub with wild olive and mastic trees	850

Table S2. Environmental characteristics measured.

**Characteristic**	**Scale and Ranks**
Altitude (m)	1 = <200, 2 = 200–400, 3 = 401–600, 4 = 601–800,5 = 801–1000 6 = >1000
Substrate	1= calcareous, 2= Numidian rock, 2= siliceous-clay, 3= clayey-sandstone, 5= marcnocalcareous, 6= numidian clays.
Exposure	1 = North, 2 = North-east, 3 = North-west, 4 = South, 5 = South-west
Slope	1: none, 2: <15%, 3:15–30%, 4: >30%
Orchidological surface (m^2)	1 = <50, 2 = 51–100, 3 = 101–300, 4 = 301–500, 5 > 500
Tree and grass cover rate (%)	1 = <5%, 2 = 5–10%, 3 = 10–25%, 4 = 25–50%, 5 = 50–100%
Pasture fire, Agricultural activities	1 = absent, 2 = minimal activity, 3 = slight activity, 4 = medium activity, 5 = very significant activity

Table S3. Biogeographical and heritage value of orchids in the Guelma province.

Code	Biogoegraphy	Included in de Bélair et al. (2005)	Rarity	Law (2012)	Walter & Gillet (1998) / IUCN (2025)/ unpublished data (Véla E., Allen D. and de Bélair G. unpublished)	Sampling stations		
						Locality name	Number of individuals (min-max)	Altitude range (min-max)
Ancf	Euro		FR	P	NE (LC probably)	9	14-150	236-340
Anpe	Med	X	FR	P	NE (LC probably)	13	5-250	236-509
Anpp	End N Alg-NW Tun-NW Ita		R*	P	EN	1	15-30	908-925
Anpl	End N Alg-N Tun		R	P	NT	2	30-85	929-1004
Celo	Med	X	R*		NT probably	1	1	819
Dael	End N-Alg NW Tun		R	P	NT	3	100-250	890-940
Hiro	Med	X	FR		LC	9	4-27	335-472
Liab	Med	X			NE (LC probably)	3	1-4	940-950
Nela	Med				NE (LC probably)	3	10-25	230-236
Nema	Med		R		NE (LC probably)	2	1-5	350-375
Opap	Med	X			NE (LC probably)	6	10-28	295-310
Opat	Betico-Magh		FR		EN	3	70-150	908-925
Opba	End Alg-Tun	X	R		NT probably	6	10-17	230-236
Opbo	Med	X			NE (LC probably)	18	12-75	236-452
Opff	Med	X			NE (LC probably)	10	10-80	236-440
Opfm	End Alg		R*		NT probably	2	7-15	650-680
Opii	Med	X			NE (LC probably)	6	14-20	215-223
Oplu	Med	X			NE (LC probably)	25	15-350	236-553
Opmc	Med		FR		NE (LC probably)	2	20-25	908-925
Opmm	Med		R*		NE (LC probably)	1	5-15	908-925
Opnu	End Alg-Tun-Mor		R		NE (LC probably)	5	90-180	908-925

Opoh	Med		R*		NE (LC probably)	1	8-15	908-925
Opsa	Med	X			NE (LC probably)	13	10-50	236-472
Opss	Med				NE (LC probably)	3	10-50	210-224
Opsp	Med	X			NE (LC probably)	25	10-250	236-620
Optf	Med	X			NE (LC probably)	15	2-15	500-509
Optt	Med	X			LC	14	5-45	210-445
Opje	End Alg-Tun		R*		NA	1	5-8	908-925
Opsu	Med		R*		NA	1	3	919
Oran	Med Atlan				NE (LC probably)	6	10-110	210-308
Orit	Med	X		P	NE (LC probably)	11	25-450	210-472
Sell	Med		R*		NT probably	3	5-12	230-236
Sels	End Alg-Tun	X	VR*		CR	1	5-10	1200
Setu	End Alg-Tun		VR*		DD	1	5-10	1200
Sepa	Med	X			NE (LC probably)	16	8-85	210-620
Sest	Med				NE (LC probably)	9	12-160	350-472
Spsp	Med				NE (LC probably)	1	1-5	540-545

X: presence, FR: fairly rare; R: rare; VR: very rare; P: protected. *Modified according to own observations. Euro: Eurosiberian, Med: Mediterranean, End: Endemic, Alg: Algerian, Tun: Tunisian, Mor: Morocco, Magh: Maghreb, Ita: Italian, N-W: Northwest, NT: Near Threatened, EN: Endangered, CR: Critically Endangered, LC: Least Concern, NE: Not Evaluad, NA: Not Applicable, DD: Insufficient Data.

Table S4. Orchid diversity and values of the variables considered at each sampling station

Station code	Taxa	Alt.	Substr.	Expos.	Slope	Orchid. Surf.	Tree cover	Grass cover	Pasture-fire	Agriculture
Boah	Ancf; Anpe; Opba; Opbo; Oplu; Opsa; Opsp; Orit; Sepa	2	6	1	1	5	2	3	3	2
Chmo	Anpe; Nema; Oplu; Opsa; Opsp; Sepa; Sest	2	4	3	1	5	2	2	2	1
Gabo	Anpe; Oplu; Opsp	2	6	4	1	2	1	2	2	3
Hoa1	Anpe; Oplu; Opsp; Orit; Sepa	2	4	4	1	1	1	2	6	1
Hoa2	Liab; Oplu; Opsp	3	6	2	2	1	2	1	5	2
Meam	Oplu	2	8	5	1	2	1	2	2	1
Ungu	Opap	2	2	1	3	2	1	1	4	2
Cofa	Hiro	3	2	1	1	2	3	2	1	1
Zsab	Opbo; Oplu; Opsa; Opsp; Optf; Oppt	4	2	1	1	5	1	3	2	1
Msms	Anpe; Anpl; Dael; Nela; Oplu; Opnu; Opsp; Optf; Oppt; Sell	6	2	4	2	6	1	4	2	2
Oufr	Opbo; Opsp; Optf; Optt	1	1	2	1	1	2	2	3	1
Bsba	Spsp	2	2	1	2	1	1	1	5	2
Atah	Opap; Opbo; Opff; Opsp; Optf; Orit; Sepa	2	6	2	2	1	2	2	5	3
Boce	Anpe; Opba; Opbo; Opff; Oplu; Opsa; Opsp; Oran; Orit	2	4	2	1	5	2	4	1	1
Race	Ancf; Anpe; Hiro; Opbp; Opii; Oplu; Opmc; Opsp; Optt; Oran; Orit; Sell; Sepa; Sest	2	4	3	1	5	2	4	1	1
Mrce	Ancf; Anpe; Nela; Opba; Opff; Opii; Oplu; Opsa; Optf; Optt; Orit; Sepa	2	4	3	1	4	1	4	1	1
Oube	Celo; Hiro; Liab	5	6	2	2	6	4	1	2	1
Mece	Hiro; Opap; Opbo; Opff; Oplu; Opsa; Optt; Orit	2	4	3	1	4	1	4	1	1

Mnbl	Anpe; Hiro; Opbo; Opff; Opii; Opsa; Opss; Opsp; Optt; Orit; Sepa; Sest	2	4	3	1	2	1	3	4	1
Ouch	Opff; Oplu; Opsp; Optf; Optt; Orit	4	5	2	2	1	1	2	4	2
Dhso	Ancf; Opbo; Oplu; Opso; Sepa	2	4	5	3	2	1	2	5	2
Nodh	Opff; Opii; Oplu	2	2	1	3	2	1	1	4	2
Aisa	Opbo; Oplu; Opsa; Opsp; Optf; Orit; Sepa; Sest	2	2	5	2	1	1	3	4	1
Mtce	Hiro; Oplu	3	4	4	1	3	1	3	1	1
Barn	Ancf; Opbo; Opfm; Oplu; Opsp	3	4	4	2	3	1	2	6	3
Dodo	Ancf; Opnu; Optf	5	7	1	2	3	1	2	3	2
Hade	Ancf; Hiro; Opap; Opba; Opbo; Opff; Oplu; Opsa; Opss; Opsp; Oran; Sepa	2	2	5	2	5	1	3	3	2
Hama	Ancf; Hiro; Opba; Opsa; Opss; Oran	2	2	3	1	3	1	3	2	3
Rokn	Ancf; Opsp; Optf; Optt	2	1	4	2	2	2	1	5	2
Djt1	Anpe; Anpp; Nema; Opap; Opat; Opbo; Opff; Oplu; Opnu; Opsa; Optf; Oran; Orit; Sepa	6	2	3	2	4	1	2	5	2
Djt2	Anpe; Nela; Opat; Opba; Opnu; Opoh; Opsa; Opsp; Optf; Sepa; Sest; Opje	6	2	3	2	4	1	2	5	2
Ouat	Ancf; Dael; Sepa; Sest	5	2	1	1	3	1	2	1	2
Aita	Liab; Optf	6	1	1	1	3	3	2	2	1
Ghdj	Anpe; Anpl; Opat; Opbo; Opfm; Oplu; Opmc; Opmm; Opnu; Opsa; Opsp; Optf; Optt; Oran; Opsu	5	2	1	2	4	1	3	3	2
Koud	Anpe; Opbo; Oplu; Opsp; Optf; Optt; Opsu	5	2	3	1	4	1	4	2	3
Ailo	Opii; Oplu; Opsp; Optt	4	2	2	1	2	2	2	2	3
Bsbr	Opbo; Oplu; Optt	4	2	5	1	3	1	3	1	1
Boma	Opsp	1	2	2	1	2	1	2	3	3

Maba	Dael; Opap; Optf; Optt; Sell; Sels; Sepa; Setu; Sest	6	2	1	3	3	3	4	2	1
Joce	Opbo; Oplu; Opsp; Sepa; Sest	4	2	4	2	2	1	2	1	1

Alt. : Altitude, Substr. : Substrate, Orchid. Surf. : Orchidological surface.

Table S5. Orchid diversity in the mini-hotspots (Kabylias-Numidia-Kroumiria)

**Information source**	**Region/province**	**Sector/subsector**	**Taxa number**	**Number of endemic taxa**
Present study	Guelma	C_1-K_3	37	6
de Bélair *et al.* (2005) [26]	Numidia	C_1-K_3	36	6
Bougaham *et al.* (2015) [56]	Small Kabylia	K_2	27	3
Boutabia *et al.* (2019) [30]	El Kala National Park	K_3	23	4
Hamel & Meddad-Hamza (2016) [27]	Edough peninsula	K_3	20	2
Hamel *et al.* (2017) [28]	Skikda (Collo is excluded)	K_3	18	1
Boukehili *et al.* (2018) [29]	Souk Ahras	C_1	27	6
El Mokni *et al.* (2012) [70]	Kroumiria and Mogod	Mogod montains	23	4
